# Comparison of pharmacokinetic similarity, immunogenicity, and safety of mepolizumab and BAT2606 in healthy Chinese men in a double-blinded, randomized, single-dose, three-arm parallel-group phase I trial

**DOI:** 10.3389/fphar.2025.1580883

**Published:** 2025-07-25

**Authors:** Cuiyun Li, Jixuan Sun, Jia Xu, Min Wu, Xiaojiao Li, Zhijie Liu, Qingfeng Dong, Yu Sun, Yanhua Ding

**Affiliations:** ^1^ Phase I Clinical Research Center, First Hospital of Jilin University, Changchun, Jilin, China; ^2^ Bio-Thera Solutions, Ltd., Guangzhou, Guangdong, China

**Keywords:** BAT2606 injection, biosimilar, mepolizumab, pharmacokinetics, safety, immunogenicity

## Abstract

**Objective:**

To evaluate the similarity of BAT2606 and mepolizumab, including pharmacokinetic profiles, immunogenicity, and safety, in healthy Chinese men.

**Methods:**

This randomized, double-blind, parallel three-arm, single-dose Phase I clinical study enrolled 207 subjects. All subjects enrolled in this study were randomly assigned to receive BAT2606 or mepolizumab (European-sourced Nucala [Nucala-EU] and US-sourced Nucala [Nucala-US]) at a 1:1:1 ratio. In total, 206 subjects received a subcutaneous injection of 100 mg of one of the drugs in this study.

**Results:**

The mean drug concentration–time curves were similar among the three groups. The 90% confidence intervals of the geometric mean ratios of maximum concentration and area under the curve from time 0 to infinity were within 80.00%–125.00%. There were no adverse events leading to study discontinuation or death, no serious adverse events, and no local injection-site reactions. The rates of adverse events and treatment-related adverse events were comparable among the BAT2606 (78.3% and 66.7%, respectively), Nucala-US (76.5% and 64.7%, respectively), and Nucala-EU groups (82.6% and 71.0%, respectively). The majority of treatment-related adverse events were grade 1 or 2 in severity based on Common Terminology Criteria for Adverse Events version 5.0. Anti-drug antibodies (ADAs) were detected in 4 (5.8%), 10 (14.7%), and 10 subjects (14.5%) in the BAT2606, Nucala-US, and Nucala-EU groups, respectively. All subjects with a positive ADA result were negative for neutralizing antibodies.

**Conclusion:**

BAT2606 injection was pharmacokinetically bioequivalent to Nucala-US and Nucala-EU in healthy Chinese men. BAT2606 was well tolerated, and its overall safety profile was similar to those of Nucala-US and Nucala-EU. BAT2606 had a numerically lower ADA incidence than Nucala-US and Nucala-EU.

**Clinical Trial Registration:**

https://www.clinicaltrials.gov/study/NCT05576454?term=BAT2606&rank=1.

## 1 Introduction

Asthma is typified by chronic airway inflammation, structural changes in the bronchi, and allergic reactions in the airways produced by various mediators released from activated inflammatory and immune cells (National Asthma Education and Prevention Program, 2007). Approximately 5%–10% of patients with asthma experience severe asthma. When severe asthma is not controlled by high-dose inhaled corticosteroid and long-acting beta-agonist therapy, a long-acting anticholinergic agent or a variety of biologic agents can be added (Côté et al., 2020).

Eosinophils play an important role in the development of asthma. In severe eosinophilic asthma, excess eosinophils release their specific toxic particulate contents, including eosinophil cationic proteins, eosinophil neurotoxins, eosinophil peroxidase, and major basic proteins, which can cause tissue damage (Fettrelet et al., 2021). In addition, eosinophils release several inflammatory factors that can affect airway remodeling, thereby limiting breathing and increasing the frequency of asthma attacks (Van et al., 2021; Berdnikovs, 2021). In fact, airway eosinophilic infiltration is frequent in both allergic and non-allergic asthma. Interleukin-5 (IL-5) is a powerful pro-inflammatory cytokine that binds to the α subunit of the IL-5 receptor, thereby activating a complex intracellular signaling network. The consequent stimulation of specific targets leads to eosinophil activation, maturation, survival, and migration. As previously reported, the complex relationships among chronic airway inflammation, airway hyperresponsiveness (AHR), and airway remodeling play important roles in the pathogenesis of asthma (Montuschi and Barnes, 2011). Effective management of chronic airway inflammation is crucial in the treatment of asthma, along with measures to reduce AHR and prevent airway remodeling. Numerous pre-clinical studies found that IL-5 can induce eosinophilia, prolong the survival of eosinophils, trigger eosinophilic inflammation, and enhance AHR. In addition, IL-5 receptors are also expressed on various cell types, including lung fibroblasts, suggesting a potential role for anti-IL-5 pharmacotherapies in airway remodeling (Bajbouj et al., 2023).

Multiple clinical trials reported that IL-5 levels and eosinophil counts are higher in patients with severe asthma and positively correlated with disease severity (Pelaia et al., 2019). Therefore, IL-5 has become a major therapeutic target for severe eosinophilic asthma.

Mepolizumab, developed and marketed by the British pharmaceutical company GlaxoSmithKline (London, United Kingdom) under the brand name Nucala^®^, is a humanized recombinant immunoglobulin G anti-human IL-5 monoclonal antibody. Mepolizumab can specifically bind IL-5 and inhibit eosinophil production, thereby exerting a therapeutic effect (FDA). Mepolizumab was first approved by the European Medicines Agency and the US Food and Drug Administration in late 2015 for the additional maintenance treatment of patients aged 12 years and older with severe eosinophilic asthma.

BAT2606 is a mepolizumab biosimilar developed by Bio-Thera Solutions, Ltd. (Guangzhou, China). The physical and chemical characteristics of BAT2606 are similar to those of mepolizumab based on the results of a structural identification study. In addition, the results of preclinical assessments of *in vitro*/*in vivo* pharmacodynamics, single-dose pharmacokinetics, multiple-dose toxicity assays, and immunogenicity revealed that the biological activity, pharmacokinetics, safety, and immunogenicity of BAT2606 were highly similar to those of mepolizumab under equivalent experimental conditions.

To obtain marketing approval for BAT2606, a subcutaneous single-dose pharmacokinetic study was conducted in healthy Chinese men to evaluate bioequivalence between BAT2606 and mepolizumab (EMA, 2014; F DA, 2016; NMPA, 2015). In addition, the tolerability, safety, and immunogenicity of BAT2606 were assessed. The results of this Phase I study also provided evidence for the Phase III study investigating the efficacy and safety of BAT2606 in the indicated patients.

## 2 Materials and methods

### 2.1 Drugs for evaluation

BAT2606 injection (specification: 100 mg/1 mL/syringe), a recombinant human IL-5 monoclonal antibody solution, was manufactured by Bio-Thera Solutions, Ltd. European-sourced Nucala^®^ and America-sourced Nucala^®^ (hereafter termed Nucala-EU and Nucala-US, respectively; specification: 100 mg/1 mL/syringe) were manufactured by GlaxoSmithKline Trading Services Limited and GlaxoSmithKline LLC, respectively.

### 2.2 Subjects

Healthy Chinese men aged 18–55 years old with a body weight of 55–85 kg and a body mass index (BMI) of 18–28 kg/m^2^ were recruited for this study. The suitability of the subjects was based on a review of the following items: medical history, physical examination, vital signs, 12-lead electrocardiography, x-ray, color Doppler ultrasound of the abdomen, blood chemistry, hematology, urinalysis, coagulation function, tuberculosis enzyme-linked immune SPOT test, serum pregnancy test, hepatitis B and C tests, and HIV diagnostic profiles. Volunteers were excluded from this study for the following reasons: an active infection within 4 weeks prior to screening; prior receipt of mepolizumab (or its biosimilar), any biologics targeting IL-5 (including the IL-5 receptor), or any biologics or monoclonal antibodies within 6 months (or within five half-lives of the medication, whichever was longer) prior to screening; and participation in other clinical drug studies within 3 months prior to enrollment.

The protocol was approved by the Research Ethics Board of the First Hospital of Jilin University (Jilin, China). This Phase I study was performed between September 2022 and June 2023 at the Phase I Clinical Trial Unit of the First Hospital of Jilin University. This study was conducted in accordance with the Declaration of Helsinki, and the protocol followed the principles of Good Clinical Practice. All recruited volunteers provided written informed consent. The clinical trial registration number is NCT05576454 (ClinicalTrials.gov).

### 2.3 Design of study and drug administration

This randomized, double-blinded, single-dose, three-arm parallel group clinical trial evaluated the pharmacokinetics, immunogenicity, and safety of BAT2606 injection compared with mepolizumab (Nucala-EU and Nucala-US) in healthy Chinese men.

The sample size was estimated using PASS version 15.0 software (NCSS, Kaysville, UT, United States) under the following settings: coefficient of variation (CV) of 0.30 (Shabbir et al., 2020), expected geometric mean ratio (GMR) of 0.95, significance level of α = 0.05 (two one-sided tests), test power of 1 − β = 0.928 (ensuring total power of 80% for three pairwise comparisons), 90% confidence intervals (CIs) within a two-sided equivalence bound of 80.00%–125.00% for area under the curve from time 0 to infinity (AUC  0−∞ ) and maximum concentration (C_max_), and a dropout rate of 15%. Considering these criteria, at least 69 subjects needed to be enrolled in a single arm. Therefore, at least 207 subjects were targeted for recruitment into the three arms of the trial.

All subjects enrolled in this study were randomly assigned at a 1:1:1 ratio to receive a subcutaneous injection of 100 mg of BAT2606, Nucala-EU, or Nucala-US. Given that this was the first clinical study of BAT2606, this pharmacokinetic comparison study was performed as a sentinel two-stage dosing study.

In stage 1, six subjects were enrolled and randomized at a 1:1:1 ratio to receive BAT2606, Nucala-EU, or Nucala-US, and the subjects were required to remain at the study site for at least 144 h (7 days) after dosing and undergo close monitoring. Ten days after dosing in this stage, the safety and tolerability of the study drugs were reviewed. If there were no serious or unexpected drug-related safety issues, the next stage of dosing was initiated. In stage 2, 201 subjects were enrolled and randomized at a 1:1:1 ratio using weight as a stratification factor, with every 10 kg of body weight as a grouping interval (≥55.0 to <65.0 kg, ≥65.0 to <75.0 kg, and ≥75.0 to ≤85.0 kg). Then, the subjects were randomized to receive BAT2606, Nucala-EU, or Nucala-US, and they were not allowed to leave the study site until 144 h (7 days) after dosing. Subsequently, the subjects returned to the site for safety and pharmacokinetic evaluations on days 8, 9, 10, 15, 22, 29, 43, 57, 71, 85, 99, and 113.

### 2.4 Pharmacokinetic assessment

To evaluate the pharmacokinetic profiles of the participants, approximately 3.5 mL of whole blood were collected in a vacuum blood collection tube at the following timepoints: 0 (pre-dose), 2, and 8 h and 2, 3, 4, 5, 6, 7, 8, 9, 10, 15, 22, 29, 43, 57, 71, 85, 99, and 113 days. After 0.5–1 h at room temperature, blood samples were centrifuged at 2°C–8°C for 15 min at 1,500–2000 × *g*. The serum samples were stored at −60°C until the drug concentration was measured using a validated enzyme-linked immunosorbent assay. The lower limit of quantification of the assay was 150 ng/mL for serum BAT2606 or mepolizumab, and the calibration range was 150–9,600 ng/mL. The accuracy ranged 0.5%–4.0%, with the precision within the 8% CV. Pharmacokinetic evaluations included the following parameters: C_max_, time to C_max_ (T_max_), AUC time 0 to the last measurable time point at which the concentration was measured (AUC_0–t_), AUC_0-∞_, AUC from T_max_ to infinity (AUC_Tmax-_  ∞ ), elimination half-life, total clearance rate (CL/F), and apparent volume of distribution. Pharmacokinetic parameters were measured using WinNonlin software (version 8.4; Pharsight Corporation, St. Louis, MO, United States) and the non-compartment model.

Descriptive analyses were also performed to assess the relationship between pharmacokinetic and pharmacodynamic parameters (eosinophils).

### 2.5 Safety evaluation

The safety of BAT2606, Nucala-EU, and Nucala-US was assessed in all treated subjects. Safety was evaluated by adverse events (AEs), local injection-site reactions, symptoms, clinical laboratory tests (blood chemistry, hematology, urinalysis, coagulation function), vital signs, physical examinations, and 12-lead electrocardiography. In this study, AEs were collected from initial drug dosing. AEs were graded according to Common Terminology Criteria for Adverse Events (CTCAE) version 5.0. Safety analyses focused on the incidence and severity of AEs, serious AEs, and other events.

### 2.6 Immunogenicity evaluation

Anti-drug antibodiy (ADA) tests were performed at 0 h (before drug dosing) and on days 10, 15, 29, 57, 85, and 113. If the subject had a positive ADA result, a neutralizing antibody (NAb) assay was performed. ADAs and NAbs that reacted to BAT2606 or mepolizumab in human serum were determined by an electrochemiluminescence assay. Samples were acid-hydrolyzed and incubated with biotin–drug and tag–drug (containing a high IL-5 receptor concentration) to form ‘drug–ADA–drug’ complexes. The instrument signal was read on the MESO QUICKPLEX SQ120. The magnitude of the instrument signal value was proportional to the ADA concentration.

### 2.7 Statistical analyses

The serum concentration *versus* time profiles of BAT2606, Nucala-EU, and Nucala-US were summarized by treatment group. Pharmacokinetic parameters were calculated using WinNonlin software with the non-compartment model. AUC_0-t_, AUC_0-∞_, AUC_Tmax-∞_, and C_max_ were log-transformed and evaluated using analysis of variance (ANOVA). The general linear model included the effects of treatment and weight. The parameters obtained from the aforementioned model were back-transformed from log scale to calculate GMRs (BAT2606 vs. Nucala-EU, BAT2606 vs. Nucala-US, and Nucala-EU vs. Nucala-US) with the 90% CIs for the relevant pharmacokinetic parameters. BAT2606 was to be considered bioequivalent to Nucala-EU and Nucala-US if the 90% CI of the GMRs of C_max_ and AUC_0-∞_ fell between 80.00% and 125.00% in the three groups in pairwise comparisons.

## 3 Results

### 3.1 Demographics

In total, 207 subjects were enrolled and randomized in this study. Of them, 200 subjects completed the study, including 69 subjects in the BAT2606 group, 65 subjects in the Nucala-US group, and 66 subjects in the Nucala-EU group. One subject (0.5%) in the Nucala-US group withdrew from the study prior to study drug administration. Three subjects each in the Nucala-US and Nucala-EU groups discontinued from the study after study drug administration. The reasons for withdrawal from the study were loss to follow-up (n = 5) and withdrawal of consent (n = 2). The demographics of the subjects were similar across the groups in terms of age, weight, and BMI, as summarized in Table 1. The number of subjects in the same weight stratification was evenly distributed among the groups.

**TABLE 1:** Demographic data of the study subjects.

Variable	BAT2606 injection (N = 69)	Nucala-US (N = 68)	Nucala-EU (N = 69)	Total (N = 206)
Age (years), mean (SD)	39.4 (9.97)	40.3 (9.53)	38.4 (10.7)	39.4 (10.1)
Body weight (kg), mean (SD)	69.2 (8.00)	69.0 (7.83)	68.8 (8.47)	69.0 (8.07)
Body height (cm), mean (SD)	168 (6.05)	168 (5.39)	169 (5.26)	169 (5.57)
BMI (kg/m^2^), mean (SD)	24.4 (2.44)	24.4 (2.28)	24.0 (2.43)	24.2 (2.38)
Race, *n* (%)				
Han	65 (94.2)	65 (95.6)	65 (94.2)	195 (94.7)
Other	4 (5.8)	3 (4.4)	4 (5.8)	11 (5.3)
Body weight, *n* (%)				
≥55.0 kg and <65.0 kg	23 (33.3)	24 (35.3)	23 (33.3)	70 (34.0)
≥65.0 kg and <75.0 kg	28 (40.6)	28 (41.2)	28 (40.6)	84 (40.8)
≥75.0 kg and <85.0 kg	18 (26.1)	16 (23.5)	18 (26.1)	52 (25.2)

SD, standard deviation; BMI, body mass index.

### 3.2 Pharmacokinetic analysis

**FIGURE 1:**
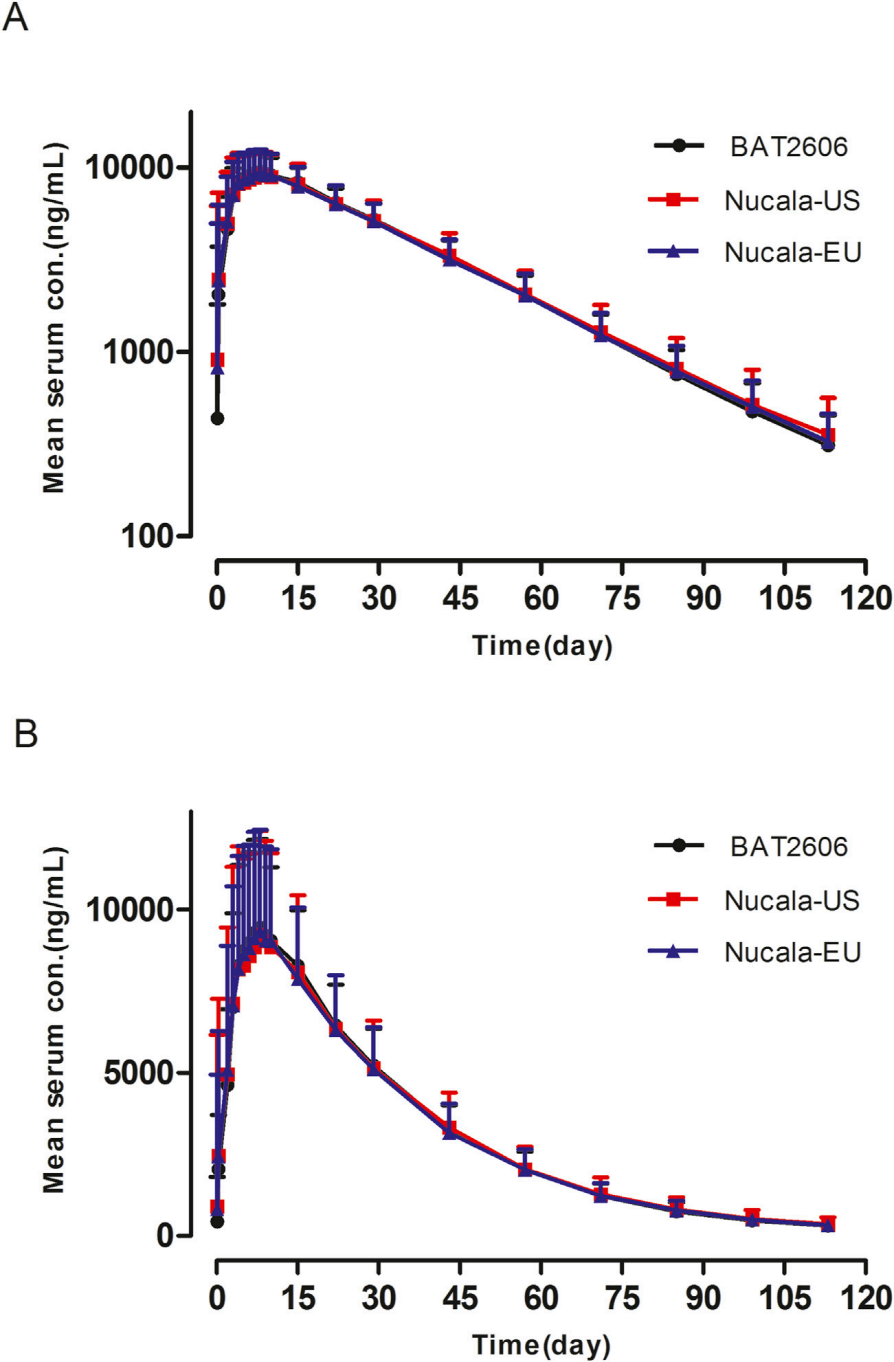
Mean serum concentration–time profiles after a single 100-mg subcutaneous dose of BAT2606, Nucala-EU, and Nucala-US. **(A)** Semi-log scale. **(B)** Linear scale.

Pharmacokinetic analysis was performed using the pharmacokinetic concentration analysis set (PKCS). Pharmacokinetic parameters and PK similarity analyses were performed using the pharmacokinetic parameter set (PKPS). The PKCS and PKPS included 206 and 203 subjects, respectively. As presented in Figure 1, the mean serum drug concentration–time curves through the scheduled time points (both linear and semi-logarithmic) were similar among the three groups. A summary of the serum pharmacokinetic parameters is presented in Table 2, revealing similar findings among the three groups. Specifically, T_max_ and the elimination half-life (t_1/2_) did not significantly differ among the groups (P > 0.05).

**TABLE 2:** Summary of the pharmacokinetic parameters after a single 100-mg subcutaneous administration of the test drug BAT2606 Injection and reference drug mepolizumab (Nucala-EU, and Nucala-US).

Pharmacokinetic parameters	BAT2606 Injection (N = 69)	Nucala-US (N = 66)	Nucala-EU (N = 68)
T_max_ (h), median (range)	168 (48–504)	168 (48–336)	168 (24–504)
C_max_ (μg/mL)	10.1 ± 2.71 (26.7%)	9.63 ± 3.08 (32.0%)	9.90 ± 3.16 (31.9%)
AUC_0-t_ (h*μg/mL)	8,500 ± 1800 (21.2%)	8,410 ± 2,400 (28.5%)	8,330 ± 2,240 (26.9%)
AUC_0-_ ∞ (h*μg/mL)	8,720 ± 1860 (21.3%)	8,670 ± 2,560 (29.5%)	8,570 ± 2,310 (27.0%)
AUC_Tmax-_ ∞ (h*μg/mL)	229 ± 139 (60.9%)	264 ± 219 (82.7%)	238 ± 138 (58.1%)
t_1/2_ (h)	489 ± 72.0 (14.7%)	497 ± 89.4 (18.0%)	489 ± 75.5 (14.3%)
Vz/F (mL)	8,390 ± 2060 (24.5%)	8,750 ± 2,290 (26.2%)	8,890 ± 3,130 (35.2%)
CLz (mL/h)	11.9 ± 2.26 (19.0%)	12.5 ± 3.48 (28.0%)	12.7 ± 4.18 (33.0%)

Data are expressed as the mean ± standard deviation (percent coefficient of variation) unless otherwise specified.

Pharmacokinetic parameters were evaluated by weight group (≥55.0 to <65.0 kg, ≥65.0 to <75.0 kg, and ≥75.0 to ≤85.0 kg), as presented in Table 3.

**TABLE 3:** Summary of the pharmacokinetic parameters after a single 100-mg subcutaneous administration of the test drug BAT2606 Injection and reference drug mepolizumab (Nucala-EU, and Nucala-US) by weight group.

Pharmacokinetic parameters	BAT2606 Injection		Nucala-US		Nucala-EU	
C_max_ (μg/mL)						
≥55.0 kg and <65.0 kg	n = 23	11.7 (18.8%)	n = 23	11.4 (25.7%)	n = 23	12.4 (18.3%)
≥65.0 kg and <75.0 kg	n = 28	9.23 (29.7%)	n = 27	9.04 (25.2%)	n = 27	9.26 (26.7%)
≥75.0 kg and ≤85.0 kg	n = 18	8.42 (24.8%)	n = 16	6.76 (33.5%)	n = 18	6.60 (39.7%)
AUC_0-_ ∞ (h*μg/mL)						
≥55.0 kg and <65.0 kg	n = 23	9,810 (19.0%)	n = 23	9,690 (27.5%)	n = 23	10,200 (19.5%)
≥65.0 kg and <75.0 kg	n = 28	8,270 (18.4%)	n = 27	8,340 (20.6%)	n = 27	8,300 (19.4%)
≥75.0 kg and ≤85.0 kg	n = 18	7,550 (12.8%)	n = 16	6,610 (27.2%)	n = 18	6,280 (30.0%)

Data are expressed as the geometric mean (geometric percent coefficient of variation).

The results of the assessment of pharmacokinetic similarity among the treatment groups are presented in Table 4. The 90% CIs of the GMRs of C_max_ and AUC_0-_  ∞ were both within 80.00%–125.00%, suggesting that BAT2606 is pharmacokinetically bioequivalent to Nucala-US and Nucala-EU.

**TABLE 4:** Statistical comparison of pharmacokinetic parameters between BAT2606 Injection and reference drug mepolizumab (Nucala-EU, and Nucala-US).

Parameter		Geometric Means		GMR with 90% CI (80.0, 125)	
	n	BAT2606 Injection	Nucala-US	Ratio (%)	90% CI (%)
C_max_ (μg/mL)	135	9.59	8.91	107.66	(99.76,116.19)
AUC_0-t_ (h*μg/mL)	134	8210	7910	103.78	(97.62,110.34)
AUC_0-∞_ (h*μg/mL)	134	8430	8140	103.52	(97.34,110.09)
AUC_Tmax-∞_ (h*μg/mL)	134	196	210	93.18	(80.14,108.34)
Parameter	n	BAT2606 Injection	Nucala-EU	Ratio (%)	90% CI (%)
C_max_ (μg/mL)	137	9.59	9.17	104.60	(96.98,112.82)
AUC_0-t_ (h*μg/mL)	135	8210	7920	103.67	(97.53,110.19)
AUC_0-∞_ (h*μg/mL)	135	8430	8150	103.46	(97.31,110.00)
AUC_Tmax-∞_ (h*μg/mL)	135	196	206	95.22	(81.95,110.64)
Parameter	n	Nucala-EU	Nucala-US	Ratio (%)	90% CI (%)
C_max_ (μg/mL)	134	9.17	8.91	102.93	(95.34,111.11)
AUC_0-t_ (h*μg/mL)	131	7920	7910	100.11	(94.10,106.51)
AUC_0-∞_ (h*μg/mL)	131	8150	8140	100.06	(94.02,106.48)
AUC_Tmax-∞_ (h*μg/mL)	131	206	210	97.86	(84.03,113.98)

CI, confidence interval.

**FIGURE 2:**
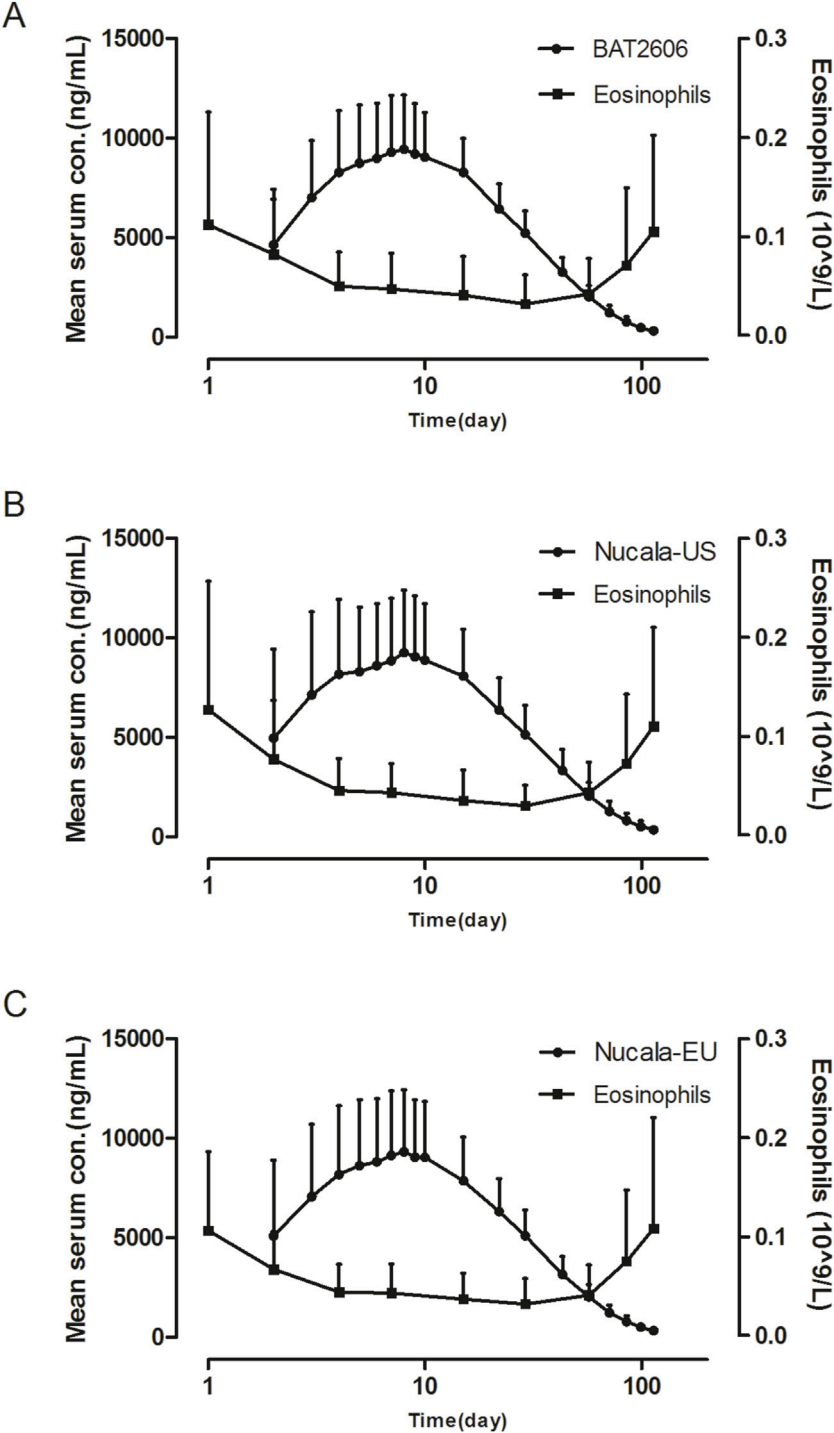
Mean serum concentration–time and eosinophil count–time profiles in the **(A)** BAT2606, **(B)** Nucala-EU, and mean Nucala-US groups **(C)** after a single 100-mg subcutaneous dose (linear scale).

The analysis of the trends of eosinophil counts *versus* serum mepolizumab concentrations by treatment group is illustrated in Figure 2. Eosinophil counts decreased as the mepolizumab concentration increased post-administration and subsequently increased as the mepolizumab concentration gradually decreased. This trend was similar in all treatment groups.

### 3.3 Assessment of drug tolerability

In total, 206 subjects were included in the safety analysis. There were no AEs leading to study discontinuation or death, no serious AEs, and no local injection-site reactions.

Overall, 163 (79.1%) subjects reported 479 AEs, including 54 subjects (78.3%) in the BAT2606 group, 52 subjects (76.5%) in the Nucala-US group, and 57 subjects (82.6%) in the Nucala-EU group. The incidence of AEs was comparable among the three groups. The common AEs (incidence ≥5%) in this study are presented in Table 5.

**TABLE 5:** Incidence of adverse events reported by ≥ 5% of subjects in any treatment group.

Adverse events	BAT2606 injection (N = 69) n (%)	Nucala-US (N = 68) n (%)	Nucala-EU (N = 69) n (%)	Overall (N = 206) n (%)
Blood triglycerides increased	34 (49.3)	28 (41.2)	24 (34.8)	86 (41.7)
Alanine aminotransferase increased	7 (10.1)	9 (13.2)	7 (10.1)	23 (11.2)
Blood creatinine increased	9 (13.0)	7 (10.3)	5 (7.2)	21 (10.2)
Aspartate aminotransferase increased	7 (10.1)	6 (8.8)	6 (8.7)	19 (9.2)
Blood uric acid increased	10 (14.5)	4 (5.9)	5 (7.2)	19 (9.2)
Protein urine present	4 (5.8)	8 (11.8)	6 (8.7)	18 (8.7)
Blood bilirubin increased	5 (7.2)	4 (5.9)	5 (7.2)	14 (6.8)
Blood glucose increased	5 (7.2)	5 (7.4)	2 (2.9)	12 (5.8)
Neutrophil count decreased	2 (2.9)	4 (5.9)	6 (8.7)	12 (5.8)
Red blood cells urine positive	5 (7.2)	4 (5.9)	3 (4.3)	12 (5.8)
Gamma-glutamyltransferase increased	4 (5.8)	6 (8.8)	1 (1.4)	11 (5.3)
Blood creatine phosphokinase increased	4 (5.8)	3 (4.4)	3 (4.3)	10 (4.9)
White blood cell count decreased	3 (4.3)	4 (5.9)	3 (4.3)	10 (4.9)
Coronavirus disease 2019	8 (11.6)	8 (11.8)	8 (11.6)	24 (11.7)
Upper respiratory tract infection	6 (8.7)	9 (13.2)	8 (11.6)	23 (11.2)

Overall, 139 subjects (67.5%) reported 315 treatment-emergent adverse drug reactions (ADRs). Similar ADRs were observed in the three groups. The most common ADRs (incidence ≥5%) in the BAT2606, Nucala-US, and Nucala-EU groups were as follows: increased blood triglycerides (46.4% vs. 39.7% vs. 33.3%), increased alanine aminotransferase (8.7% vs. 11.8% vs. 8.7%), increased aspartate aminotransferase (8.7% vs. 8.8% vs. 7.2%), increased blood creatinine (10.1% vs. 7.4% vs. 4.3%), increased blood uric acid (7.2% vs. 4.4% vs. 5.8%), presence of protein urine (4.3% vs. 7.4% vs. 5.8%), decreased neutrophil count (2.9% vs. 4.4% vs. 8.7%), increased blood bilirubin (4.3% vs. 2.9% vs. 7.2%), increased blood glucose (2.9% vs. 7.4% vs. 2.9%), and increased gamma-glutamyltransferase (5.8% vs. 4.4% vs. 0).

The overall incidence rates of grades 1, 2, and 3 ADRs (CTCAE version 5.0) were 53.9%, 30.6%, and 4.9%, respectively. The incidence rates of grade 1 ADRs in the BAT2606, Nucala-US, and Nucala-EU groups were 56.5%, 55.9%, and 49.3%, respectively, and the rates of grade 2 ADRs were 29.0%, 25.0%, and 37.7%, respectively. Only 10 grade 3 ADRs were recorded, and they were observed in 10 subjects (4.9%), including five subjects (7.2%) with increased blood triglycerides and one subject (1.4%) with increased amylase in the BAT2606 group; one subject each (1.5%) with increased blood triglycerides, decreased neutrophil counts, and ear infection in the Nucala-US group; and one subject (1.4%) with increased blood triglycerides in the Nucala-EU group. There were no grade 4 or 5 ADRs.

### 3.4 Assessment of drug immunogenicity

Twenty-four subjects (11.7%) had a positive ADA status during the study. One subject in each group was ADA-positive prior to study drug administration. After drug administration, 3 (4.3%), 10 (14.7%), and nine subjects (13.0%) in the BAT2606, Nucala-US, and Nucala-EU groups, respectively, were ADA-positive. The incidence of ADA positivity was numerically lower in the BAT2606 group than the Nucala-US and Nucala-EU groups. The NAb status of all subjects with a positive ADA result was negative.

A descriptive summary of the effects of ADA positivity on pharmacokinetic parameters is presented in Table 6. The trends in the primary pharmacokinetic parameters (C_max_ and AUC_0-_  ∞ ) of mepolizumab by ADA status were similar between the BAT2606 and Nucala-US groups, but an inverse trend was observed in the Nucala-EU group. C_max_ and AUC_0-_  ∞ among the subjects with ADA positivity were numerically lower than those of the subjects with ADA negativity in the BAT2606 and Nucala-US groups, whereas their values were higher among ADA-positive subjects in the Nucala-EU group.

**TABLE 6:** Descriptive summary of effects of positive anti-drug antibodiy (ADA) on pharmacokinetic parameters.

Pharmacokinetic parameters		BAT2606 injection (N = 69)		Nucala-US (N = 66)		Nucala-EU (N = 68)	
		ADA positive	ADA negative	ADA positive	ADA negative	ADA positive	ADA negative
C_max_ (μg/mL)	n	4	65	10	56	10	58
	Geometric Mean (CV)	9.48 (13.1%)	9.78 (29.3%)	7.93 (38.4%)	9.38 (33.1%)	10.6 (39.7%)	9.14 (37.4%)
AUC_0-t_ (h*μg/mL)	n	4	65	10	55	9	57
	Geometric Mean (CV)	7,770 (8.05%)	8,360 (20.7%)	6,730 (34.4%)	8,380 (25.6%)	8,660 (27.9%)	7,910 (30.2%)
AUC_0-_ ∞ (h*μg/mL)	n	4	65	10	55	9	57
	Geometric Mean (CV)	7,910 (8.33%)	8,590 (20.5%)	6,900 (34.3%)	8,630 (26.2%)	8,910 (27.5%)	8,140 (29.9%)
AUC_Tmax-_ ∞ (h*μg/mL)	n	4	65	10	55	9	57
	Geometric Mean (CV)	138 (30.6%)	204 (55.3%)	162 (46.5%)	227 (66.7%)	227 (54.3%)	206 (53.5%)

Abbreviations: CV, coefficient of variation; n = number.

Furthermore, there was no significant difference in the mean serum concentration–time curves between the ADA-positive subjects and all participants among the three treatment groups. ADA-positive subjects were excluded from the sensitivity analysis. After the exclusion of ADA-positive subjects, the 90% CIs of the GMRs of C_max_ and AUC_0-_  ∞ were within 80.00%–125.00%, reflecting an acceptable range of pharmacokinetic similarity.

## 4 Discussion

This randomized, double-blind, parallel three-arm, single-dose Phase I clinical study evaluated the similarity of BAT2606 and mepolizumab, including pharmacokinetic profiles, immunogenicity, and safety.

BAT2606 was safe and well tolerated, and its safety profile was comparable to those of the reference drugs (Nucala-US and Nucala-EU) in terms of the incidence and severity of AEs. Most AEs were mild and manageable. No AEs leading to study discontinuation or death, no serious AEs, and no local injection-site reactions were reported in this study.

Mepolizumab is absorbed slowly by the subcutaneous route, with its T_max_ reaching 168 h. The subcutaneous route offers an extra benefit because mepolizumab can be targeted to the lymphatic system, which is typified by IL-5 expression (Trevaskis et al., 2015). Mepolizumab is absorbed within days because lymph fluid drains slowly into the vascular system (Liu, 2018).

On average, t_1/2_ of mepolizumab was approximately 20 days in this study, suggesting it had an Fc region that protected it from systemic catabolism through binding to the neonatal Fc receptor, consistent with previous research (Matera et al., 2019). Mepolizumab plasma concentrations declined bi-exponentially after intravenous infusion. However, an obvious bi-exponential decline after C_max_ for subcutaneous injection of 100 mg mepolizumab was not observed (Smith et al., 2011). According to previous clinical data, body weight was a statistically significant covariate for CL/F of mepolizumab (Serra López-Matencio et al., 2018). Subgroup analysis according to body weight illustrated that body weight affected the pharmacokinetic parameters of mepolizumab, with similar trends across the three groups. These findings were consistent with previous clinical data. The 90% CIs of the GRMs of C_max_ and AUC_0-_  ∞ were within 80.00%–125.00%, suggesting that BAT2606 was pharmacokinetically bioequivalent to Nucala-US and Nucala-EU in healthy Chinese men. C_max_ occurred at the first time point (2 h) after drug administration in two subjects, including one subject each in the Nucala-US (43,545.9 ng/mL) and Nucala-EU groups (34,279.3 ng/mL), leading to a significant increase in the mean concentration for these two groups at this time point. The most likely explanation for this finding is that the drug was accidently injected into muscle or blood vessels. However, the median concentrations of the three groups were comparable at this time point.

The incidence of ADA positivity was lower in the BAT2606 group than in the other two groups. All ADA-positive subjects were negative for NAbs. Extremely small differences in pharmacokinetic profiles were observed between ADA-positive and ADA-negative subjects. However, limited by the overall small number of subjects with ADA positivity in the three groups, no significant effects of immunogenicity on the pharmacokinetics of mepolizumab were found in the study, consistent with the findings of the public assessment report (FDA, 2015).

Regarding pharmacodynamics, mepolizumab can inhibit eosinophil production by specifically binding to IL-5, thereby exerting a therapeutic effect (EMA, 2014). The results demonstrated that the three treatments similarly decreased eosinophil counts after a single subcutaneous injection, indicating a lack of an effect of immunogenicity.

No acute or delayed anaphylactic reactions developed in ADA-positive subjects, indicating the absence of clinically drug-specific immunogenicity. We observed no impact of the immunogenic responses to mepolizumab on drug safety in this study. However, it is necessary to closely monitor the immunogenicity of BAT2606 in further Phase 3 studies.

A limitation of our study was that it only enrolled male subjects. However, the healthy male population was the most sensitive population in PK biosimilarity comparison studies, and the data were more appropriate to support the clinical development of biosimilars (EMA, 2014; F DA, 2016). In addition, previous population pharmacokinetic analyses of mepolizumab indicated that sex did not impact the pharmacokinetics of mepolizumab (FDA, 2015). In addition, the influence of the female physiological cycle was excluded, thus reducing the interference of inter-subject variability on PK parameters (Schwartz, 2003). Another limitation was that a single dose was administered in this study, and the immunogenicity of the treatment might not have been fully detected.

## 5 Conclusion

BAT2606 was pharmacokinetically bioequivalent to Nucala-US and Nucala-EU in healthy Chinese men. BAT2606 was well tolerated, with comparable safety and immunogenicity profiles as Nucala-US and Nucala-EU. BAT2606 was linked to a numerically lower ADA incidence than Nucala-US and Nucala-EU. No NAbs were induced in this study. There was no obvious influence of immunogenicity on the pharmacokinetics and safety of BAT2606/Nucala^®^.

## Data Availability

The raw data supporting the conclusions of this article will be made available by the authors, without undue reservation.
